# Effectiveness of preoperative intravenous tranexamic acid on reduction of perioperative blood loss in open intramedullary nail fixation of femoral shaft fractures in a tertiary hospital, Uganda: a prospective cohort study

**DOI:** 10.1186/s12891-026-09793-y

**Published:** 2026-04-14

**Authors:** Paul Kaweesa Kabazzi, Patrick Sekimpi, Edward Kironde, Yakobo Nsubuga, Esther Mirembe

**Affiliations:** 1 Department of Orthopedic Surgery, Masaka Regional Referral Hospital, Masaka, Uganda; 2 Department of Orthopedic Surgery, Mulago National Referral Hospital, Kampala, Uganda; 3 Faculty of Medicine, Gulu University, Gulu, Uganda; 4 Department of Research and Data, SEMA, Kampala, Uganda

**Keywords:** Tranexamic Acid, Open Intramedullary Nailing, Femoral Shaft Fractures, Perioperative Blood Loss, Resource-limited Settings

## Abstract

**Background:**

Open intramedullary nailing of femoral shaft fractures (OIN) is frequently performed in low-resource settings where limited access to fluoroscopy necessitates open reduction techniques associated with substantial perioperative blood loss. In environments where blood supply is constrained and transfusion carries additional risks, strategies to minimize bleeding are critical. Tranexamic acid reduces perioperative bleeding in multiple orthopaedic procedures; however, evidence supporting its use during OIN in low- and middle-income countries remains limited.

**Methods:**

We conducted an open-label, single-arm prospective cohort study at a tertiary hospital in Uganda between December 2018 and March 2019. Adult patients undergoing OIN received a single preoperative intravenous dose of tranexamic acid (15 mg/kg). Perioperative blood loss was assessed using change in haemoglobin concentration measured preoperatively and at 72 h postoperatively, and compared with a historical institutional mean from patients managed without tranexamic acid. Secondary outcomes included blood transfusion requirements and adverse events.

**Results:**

Forty-three patients were included. The mean perioperative haemoglobin decline was 1.7 g/dL. This observed decline was lower than the historical mean of 3.31 g/dL reported in cohorts managed without tranexamic acid, corresponding to a mean difference of 1.6 g/dL (*p* < 0.001). Two patients (4.7%) required intraoperative blood transfusion, each receiving one unit of whole blood. No postoperative transfusions were administered. Minor adverse events were infrequent, and no clinically apparent thromboembolic events were identified during the follow-up period.

**Conclusions:**

Preoperative intravenous tranexamic acid was associated with reduced perioperative blood loss and low transfusion requirements during OIN in this resource-limited setting. Controlled studies are needed to confirm these findings.

**Trial registration:**

ClinicalTrials.gov NCT07261930. Retrospectively registered on 21 July 2025.

## Background

Femoral shaft fractures represent one of the most frequent and severe long bone injuries encountered in orthopedic trauma, especially among young adult males in low- and middle-income countries (LMICs) [1]. These injuries are predominantly caused by high-energy mechanisms, with road traffic accidents accounting for the majority of cases in LMICs due to increasing motorization and poor road safety enforcement [2]. Prompt surgical fixation of femoral shaft fractures improves patient outcomes by enabling early mobilization, reducing complications such as pulmonary embolism and pressure ulcers, and shortening hospital stays [3].

In high-income settings, closed intramedullary nailing performed under fluoroscopic guidance is the gold standard for femoral shaft fracture fixation. This approach is minimally invasive, associated with less perioperative blood loss, and permits precise alignment without extensive soft-tissue disruption [4, 5]. In contrast, many LMIC hospitals lack fluoroscopy, necessitating open reduction and the use of external targeting jig systems such as the Surgical Implant Generation Network (SIGN) system, which enables interlocking nail fixation without intraoperative imaging [6, 7]. Open intramedullary nailing of femoral shaft fractures (OIN) involves more extensive surgical exposure and soft-tissue dissection, often including callus release in delayed presentations, and has been associated with increased intraoperative blood loss and prolonged operative duration [8, 9]. Studies conducted in such settings have reported average intraoperative blood loss exceeding 800 to 1500 mL during OIN, with haemoglobin drops commonly surpassing 3 g/dL [8, 9]. Such levels of blood loss pose significant clinical and logistical challenges in environments where blood banks are under-resourced, supply is inconsistent, and transfusion carries risks of infection, alloimmunisation, and adverse reactions [10–12]. Moreover, delays in surgery due to blood availability can further worsen outcomes, particularly for patients who present late after injury [9].

Tranexamic acid (TXA) is an antifibrinolytic agent that inhibits plasminogen activation and stabilizes fibrin clot formation [13]. TXA has demonstrated efficacy in reducing perioperative blood loss and transfusion requirements in multiple surgical disciplines, including spine surgery, hip fracture fixation, total hip and knee arthroplasty and cardiac surgery [14]. Large-scale trauma trials have demonstrated a mortality benefit when tranexamic acid is administered early following injury, without a significant increase in vascular occlusive events [15]. In orthopaedic populations, systematic reviews and meta-analyses have similarly confirmed its safety profile, showing no significant increase in thromboembolic complications when used appropriately [14, 16–18].

Despite this robust evidence base in elective surgery, the role of TXA in trauma-related orthopedic procedures, particularly in OIN within LMICs, remains underexplored One open-label randomized trial conducted in Iran demonstrated significantly reduced haemoglobin drop and transfusion rates with preoperative TXA in patients undergoing OIN [19]. Although intramedullary nailing outcomes have been described in low- and middle-income countries, most reports focus on fracture union and complication rates rather than perioperative blood loss. Studies from sub-Saharan Africa evaluating predictors of haemoglobin decline or intraoperative blood loss in open intramedullary nailing remain limited [3, 8, 9].

This study was designed to evaluate the effectiveness and safety of a preoperative intravenous dose of TXA in reducing perioperative blood loss among adults undergoing OIN in a tertiary hospital in Uganda. The findings are intended to provide locally relevant evidence to guide blood conservation strategies, inform perioperative care protocols, and support the adoption of TXA use in comparable resource-limited surgical settings.

The primary objective was to assess the effectiveness of TXA in reducing perioperative blood loss. The secondary objective was to evaluate the safety of TXA by documenting perioperative adverse events, including thromboembolic complications. A third, exploratory objective was to describe clinical and surgical factors that appeared to be associated with perioperative haemoglobin decline in this cohort.

## Methods

We conducted an open-label, single-arm prospective cohort study between December 2018 and March 2019 at Mulago National Referral Hospital in Kampala, Uganda. Mulago is a tertiary public teaching hospital affiliated with Makerere University College of Health Sciences, serving a large trauma patient population. Due to limited access to intraoperative imaging, femoral shaft fractures at this institution are typically treated with OIN.

### Sample size calculation

The sample size was calculated assuming a two-sided significance level of 5% (Zα/2 = 1.96) and a statistical power of 80% (Zβ = 0.84). Using the standard formula for comparison of two independent means:

$$\mathrm{n}=2\left(\mathrm{Z}\upalpha/2+\mathrm{Z}\upbeta\right)^2\,\upsigma^2\,/\,\Delta^2$$

A clinically meaningful reduction in perioperative haemoglobin decline of approximately 30% was assumed, based on previously published institutional data from patients undergoing OIN without tranexamic acid, as reported by Kajja et al. [9]. The resulting minimum sample size was adjusted to account for potential non-response or incomplete follow-up, yielding a final target sample size of 43 participants.

### Participants

Eligible participants were adult patients (aged 18 years or older) with isolated, closed femoral shaft fractures scheduled for elective OIN. Patients were excluded if the time from injury exceeded one month; if the fracture was pathological; if more than one fracture required open fixation during the same operation; or if there had been previous surgery on the affected femur. Additional exclusion criteria included conditions associated with altered bleeding or coagulation risk, including diabetes mellitus, cardiovascular disease, coagulopathy, current anticoagulant use, known hypersensitivity to tranexamic acid, or a history of thromboembolic disease. Patients with severe systemic disease corresponding to American Society of Anesthesiologists (ASA) physical status class III or higher were also excluded. Written informed consent was obtained from all participants.

**Fig. 1:**
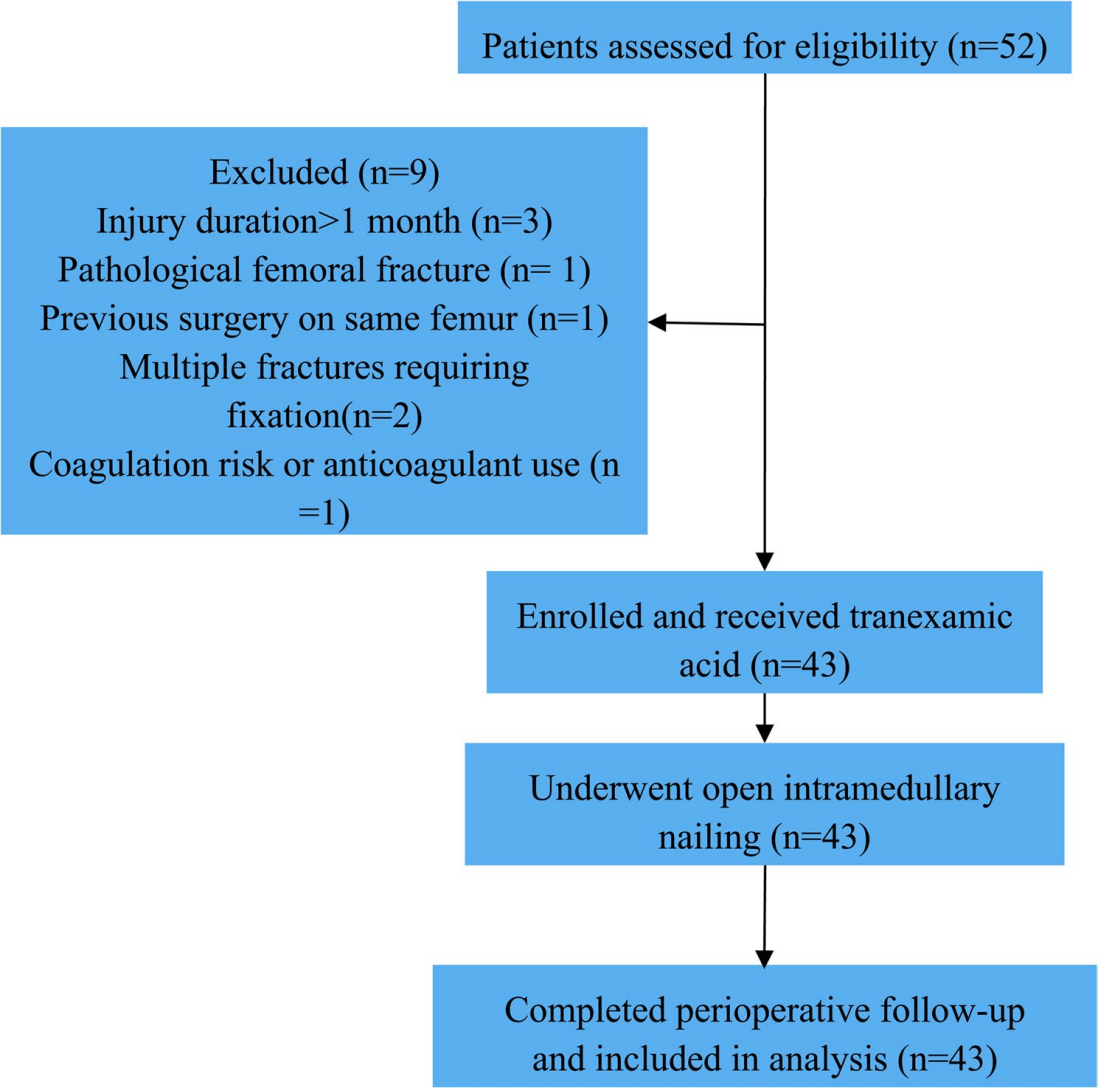
Flow diagram illustrating patient screening, exclusions, enrolment, and inclusion in the final analysis

A flow diagram illustrating patient screening, exclusions, and final inclusion is presented in Fig. 1.

### Intervention and procedures

All participants received a single preoperative dose of tranexamic acid (TXA) at 15 mg/kg body weight, administered intravenously over 10 min by the anesthesiologist approximately 10 min before skin incision. The TXA used was a generic formulation (Kapron^®^, Amoun Pharmaceutical Co., Egypt; 500 mg/5 mL ampoule). This administration protocol was selected to optimise plasma levels at the time of incision and to minimise potential side effects such as hypotension and nausea.

Preoperative care included intravenous antibiotic prophylaxis administered at induction and either general or regional anaesthesia, with spinal anaesthesia used in most cases. Surgical approach (antegrade or retrograde) was selected based on fracture location and patient anatomy.

All procedures were performed by experienced orthopaedic surgeons using external targeting jigs. The medullary canal was prepared by hand reaming through the fracture site. Cannulated interlocking intramedullary nails from three manufacturers based in India were used interchangeably during the study period: BBN^®^ Interlocking Nail System (BBN Medical Devices, India), Porto^®^ Interlocking Nail System (Porto Orthopaedics, India), and Sharma^®^ Interlocking Nail System (Sharma Orthopedic, India). All nail systems were compatible with external targeting arms and jigs and employed similar interlocking mechanisms. Interlocking screws were inserted using the target arm, with the number of interlocking bolts determined by fracture configuration and nail length.

Although the surgical technique employed external targeting jigs similar to those used in the Surgical Implant Generation Network (SIGN) system, the implants used in this study were cannulated interlocking intramedullary nails and not the solid SIGN^®^ nail system.

Preoperative planning included estimation of nail length using available radiographs and contralateral limb measurements where applicable. Final nail diameter was selected intraoperatively following hand reaming of the medullary canal.

Postoperative care included continuation of intravenous antibiotics for 72 h, parenteral analgesia, and early mobilisation. Routine pharmacologic thromboprophylaxis was not administered; thromboembolic risk was mitigated through early mobilisation and clinical monitoring.

Postoperative assessment for deep vein thrombosis was based on clinical evaluation. Routine screening with ultrasonography or D-dimer testing was not performed, and further diagnostic investigations were undertaken only when clinically indicated.

Patients were encouraged to begin mobilisation as tolerated from the first postoperative day, with weight-bearing guided by fracture stability, fixation construct, and surgeon’s discretion.

### Primary outcome: change in haemoglobin concentration (g/dL) as a surrogate for blood loss

Perioperative haemoglobin change has been used as a surrogate measure of blood loss in orthopaedic and perioperative research [9, 20, 21].

Preoperative haemoglobin concentration was measured two hours before surgery, and postoperative haemoglobin concentration was measured at 72 h after surgery. Perioperative blood loss was quantified as the net haemoglobin decline, adjusted for transfused units, using the following calculation:

$$\begin{aligned}&Preoperative\,haemoglobin-postoperative\,haemoglobin\\&+(number\,of\,transfused\,units\times1\,g/dL)\end{aligned}$$

The observed perioperative haemoglobin change in the study cohort was subsequently examined in relation to a previously published institutional mean derived from patients who underwent open intramedullary nailing without tranexamic acid.

Secondary outcomes:

- The proportion of patients requiring allogeneic blood transfusion.
- Any adverse events related to tranexamic acid (e.g., hypotension, nausea, vomiting, thromboembolic events).

Blood transfusion decisions followed predefined hospital protocols. Intraoperative transfusion was considered based on estimated blood loss, hemodynamic instability, and haemoglobin levels≤ 7 g/dL. Postoperative transfusion was indicated if haemoglobin was ≤ 6 g/dL or if clinical signs of hypoperfusion were present. One unit of whole blood (≈ 450 mL) was assumed to raise haemoglobin by approximately 1 g/dL.

### Statistical analysis

Data were entered and analyzed using Stata version 18 (StataCorp LLC, College Station, TX). Continuous variables were summarized using means and standard deviations (SD) or medians and interquartile ranges, and categorical variables using frequencies and percentages. To test our hypothesis that tranexamic acid would reduce haemoglobin loss by at least 30% relative to historical data, we used a one-sample t-test to compare the observed mean haemoglobin drop to a historical mean of 3.31 g/dL, previously reported in patients undergoing OIN without tranexamic acid [9]. Bivariate analysis used chi-square or Fisher’s exact test for categorical variables, and Student’s t-test or Wilcoxon rank-sum test for continuous variables.

Multivariable linear regression was performed to identify independent predictors of perioperative blood loss. These regression analyses were considered exploratory and were not powered as primary study endpoints.

## Results

### Baseline characteristics

A total of 43 patients underwent OIN for isolated femoral shaft fractures and received intravenous tranexamic acid. The majority of patients were male (38, 88.4%), and the mean age was 37 years (range: 19–80 years). The mean weight was 74.8 kg (range: 56–150 kg). The most common fracture pattern was the Arbeitsgemeinschaft für /Osteosynthesefragen / Orthopaedic Trauma Association (AO/OTA type A). The mean preoperative haemoglobin concentration was 12.9 ± 1.5 g/dL (Table 1).

**Table 1:** Clinical-demographic characteristics

Variable	Frequency	Percentage %
Sex		
Male	38	88.4
Female	5	11.6
Age Category		
<=20	4	9.3
21–30	17	39.5
31–40	8	18.6
41–50	7	16.3
> 50	7	16.3
Fracture pattern		
A	29	67.4
B	12	27.9
C	2	4.7
Pre-op Haemoglobin	12.9	± 1.5

Regional (spinal) anaesthesia was used in 39 patients (90.7%), and general anaesthesia in the remaining 4 patients. Antegrade nailing was performed in 38 cases (88.4%) and retrograde in 5 (11.6%). Only 2 patients (4.7%) required blood transfusion, and both received a single unit of whole blood intraoperatively. The average surgical duration was 1.7 h (range: 0.8–3.3 h) (Table 2).

**Table 2:** Summary of Surgery Variables

Variable	Frequency	Percentage %
Type of Anesthesia		
General	4	9.3
Regional	39	90.7
Nailing approach		
Antegrade	38	88.4
Retrograde	5	11.6
Patients transfused		
Yes	2	4.7
No	41	95.3

### Perioperative blood loss

The mean perioperative haemoglobin decline in the study cohort was 1.7 g/dL. This value was lower than the mean haemoglobin decline of 3.31 g/dL previously reported by Kajja et al. in similar patients undergoing OIN without tranexamic acid [9].

Using a one-sample t-test, the observed haemoglobin decline was lower than the historical comparator, and the difference was statistically significant (mean difference 1.6 g/dL, *p* < 0.001) (Table 3).

**Table 3:** One sample student t test

Variable	Mean	Hypothesized Mean	Mean difference	t statistic	*p*-value
Perioperative haemoglobin decline (g/dL)	1.7	3.31	1.6	-16.3	< 0.001

### Blood transfusion outcomes

Two patients (4.65%) required transfusion, both intraoperatively. No postoperative transfusions were administered. Each transfused patient received only one unit of whole blood, and both met standardized criteria for intraoperative transfusion.

### Safety and adverse events

Nine patients (20.9%) experienced minor adverse events following administration of tranexamic acid. The most commonly observed event was transient hypotension, accounting for five cases. Other reported events included dizziness and nausea. No thromboembolic events were observed (Table 4).

**Table 4:** Adverse events following tranexamic acid administration (*n* = 43)

Adverse event	Number of patients	Percentage (%)
Transient hypotension	5	11.6
Dizziness	2	4.7
Nausea	2	4.7
Deep vein thrombosis	0	0
Pulmonary embolism	0	0
Other thromboembolic events	0	0

Thromboembolic events were assessed clinically during hospital stay and up to two weeks postoperatively. Routine screening ultrasonography was not performed

### Exploratory analyses: clinical and surgical factors associated with perioperative haemoglobin decline

**Fig. 2:**
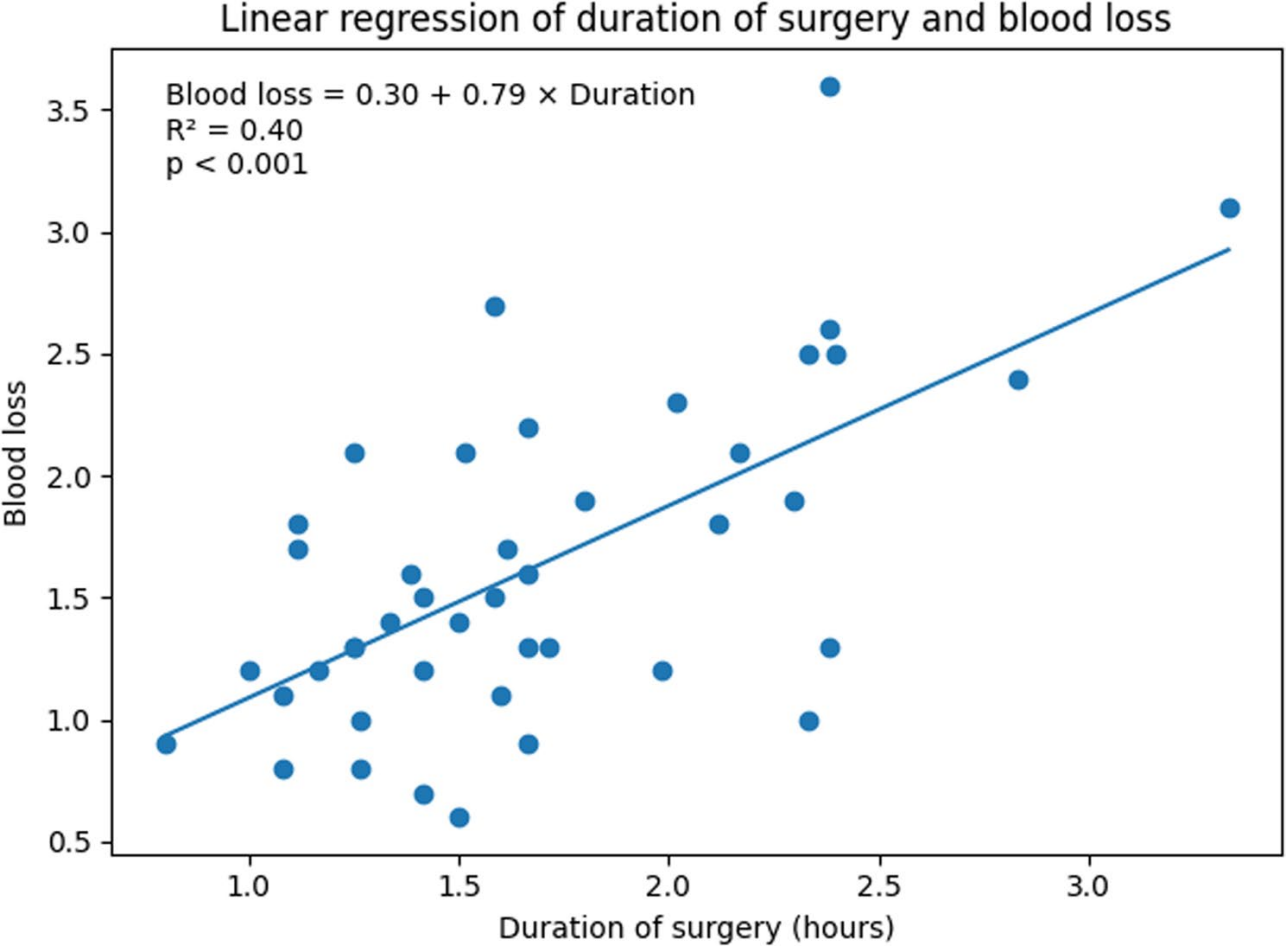
Scatter plot illustrating the relationship between operative duration and perioperative haemoglobin decline

On multivariable linear regression analysis, longer time from injury to surgery and longer operative duration appeared to be associated with increased perioperative haemoglobin decline (Table 5). Each additional day from injury to surgery was associated with an increase in haemoglobin loss of approximately 0.03 g/dL (*p* < 0.001), and each additional hour of operative time was associated with an increase of approximately 0.66 g/dL (*p* < 0.001). The relationship between operative duration and perioperative haemoglobin decline is illustrated in Fig. 2.

**Table 5:** Multivariable analysis of clinical and surgical factors associated with perioperative haemoglobin decline

Variable	Perioperative haemoglobin decline (g/dL)			
	Coef (95% CI)	*P*-value	Adjusted Coef (95% CI)	*P*-value
Sex				
Male	Ref		Ref	
Female	-0.32 (-0.92, 0.28)	0.288	-0.23 (-0.58, 0.11)	0.173
Age				
<=20	Ref			
21–30	-0.05 (-0.74, 0.64)	0.888		
31–40	-0.52 (-1.29, 0.24)	0.172		
41–50	0.08 (-0.71, 0.86)	0.847		
> 50	-0.35 (-1.13, 0.43)	0.365		
Age	0 (-0.01, 0.01)	0.562		
Weight	0.01 (-0.01, 0.02)	0.242		
Duration of Injury in days	0.04 (0.02, 0.06)	< 0.001	0.03 (0.02, 0.04)	< 0.001
Duration of Surgery in hours	0.78 (0.5, 1.05)	< 0.001	0.66 (0.45, 0.87)	< 0.001
Fracture pattern				
A	Ref		Ref	
B	0.09 (-0.33, 0.51)	0.667	-0.04 (-0.29, 0.2)	0.717
C	0.96 (0.06, 1.85)	0.037	0.55 (0.02, 1.08)	0.043
Type of Anesthesia				
General	Ref			
Regional	0.15 (-0.52, 0.82)	0.649		
Nailing Approach				
Antegrade	Ref			
Retrograde	0.13 (-0.47, 0.74)	0.661		
Preop Haemoglobin	0.02 (-0.1, 0.15)	0.691		

Multivariable linear regression model examining clinical and surgical factors associated with perioperative haemoglobin decline. Variables were included based on clinical relevance. Results should be interpreted as exploratory

Fracture pattern also appeared to be associated with haemoglobin decline, with AO/OTA type C fractures demonstrating greater haemoglobin loss compared with type A fractures (*p* = 0.043).

## Discussion

This prospective single-arm cohort study evaluated preoperative intravenous tranexamic acid in adults undergoing OIN in a resource-limited setting. We observed reduced perioperative blood loss and low transfusion requirements.

The mean perioperative blood loss was lower than previously reported at the same institution [9]. Although the absence of a control group limits causal inference, the observed difference aligns with previous orthopaedic studies [16, 19, 22].

The proportion of patients requiring blood transfusion in this study was low (4.65%), with all transfusions limited to a single unit administered intraoperatively according to predefined criteria. This transfusion rate was lower than that reported in comparable settings without tranexamic acid use, such as the 27.5% observed by Kajja et al. [9]. Reduced transfusion requirements with tranexamic acid have been reported across a range of orthopaedic procedures, including hip fracture surgery and trauma-related operations [19, 22, 23]. All transfused patients received a single unit of blood in accordance with predefined transfusion criteria.

Most evidence on tranexamic acid originates from high-resource settings. In contrast, OIN remains common in LMICs, where greater surgical exposure may increase blood loss [3, 7]. The findings of this study suggest that tranexamic acid may also have utility in such settings [24]. Tranexamic acid is inexpensive, widely available, and easy to administer, making it a practical adjunct in resource-limited environments [14].

No clinically apparent thromboembolic events were identified during the two-week follow-up period, and adverse events potentially attributable to tranexamic acid were infrequent and mild. These findings are consistent with existing literature suggesting that tranexamic acid is generally safe when used in orthopaedic surgery [14, 16]. However, thromboembolic surveillance was based on clinical assessment rather than routine imaging, and the short follow-up period may not have captured late or asymptomatic events.

As an exploratory objective, selected clinical and surgical factors were examined for possible associations with perioperative blood loss. Longer time from injury to surgery, increased operative duration, and more complex fracture patterns appeared to be associated with greater blood loss. Similar associations have been described in previous studies of femoral fracture surgery, where delayed fixation and prolonged operative time have been linked to increased bleeding due to more extensive dissection and tissue handling [8, 9]. These findings should be interpreted cautiously, as the study was not powered to evaluate these factors as primary outcomes.

This study provides context-specific data from a high-volume trauma centre in a LMIC, supporting further controlled studies to define the role of tranexamic acid in similar settings.

## Limitations

This study employed a single-arm design, limiting causal inference in the absence of a control group. Comparisons with previously published data may be influenced by heterogeneity in patient, fracture, and surgical characteristics, including differences in age, injury mechanism, fracture comminution, surgical approach, extent of exposure, and reaming technique.

Follow-up for thromboembolic events was limited to two weeks and based on clinical assessment without routine imaging, which may have reduced detection of asymptomatic events. The modest sample size and short follow-up may have limited detection of uncommon or late complications. These limitations should be considered when interpreting the generalizability of the findings to other clinical settings.

## Conclusions

Preoperative intravenous tranexamic acid was associated with reduced perioperative blood loss and low transfusion requirements during OIN in this resource-limited setting. Given the absence of a control group and modest sample size, randomized controlled trials are needed to confirm these findings.

## Data Availability

All data generated or analysed during this study are included in this published article and its supplementary information files. Additional datasets are available from the corresponding author on reasonable request.

## Abbreviations

AO/OTA: Arbeitsgemeinschaft für Osteosynthesefragen / Orthopaedic Trauma Association
ASA: American Society of Anesthesiologists
DVT: Deep vein thrombosis
Hb: Haemoglobin
TXA: Tranexamic acid
OIN: Open intramedullary nailing of femoral shaft fractures
LMICs: Low-and middle-income countries
SIGN: Surgical Implant Generation Network
SOMREC: Makerere University School of Medicine Research and Ethics Committee
